# Diverse Surface Chemistry of Cobalt Ferrite Nanoparticles to Optimize Copper(II) Removal from Aqueous Media

**DOI:** 10.3390/ma13071537

**Published:** 2020-03-27

**Authors:** Kosmas Vamvakidis, Theodora-Marianna Kostitsi, Antonis Makridis, Catherine Dendrinou-Samara

**Affiliations:** 1 Department of Chemistry, Aristotle University of Thessaloniki, 54124 Thessaloniki, Greece; kvamvaki@chem.auth.gr (K.V.); tia.marian92@gmail.com (T.-M.K.); 2 Department of Physics, Aristotle University of Thessaloniki, 54124 Thessaloniki, Greece; anmakrid@physics.auth.gr

**Keywords:** magnetic nanoparticles, nanoadsorbents, surface modification, copper(II) adsorption

## Abstract

Water pollution by heavy metals is one of the most serious worldwide environmental issues. With a focus on copper(II) ions and copper complex removal, in the present study, ultra-small primary CoFe_2_O_4_ magnetic nanoparticles (MNPs) coated with octadecylamine (ODA) of adequate magnetization were solvothermally prepared. The surface modification of the initial MNPs was adapted via three different chemical approaches based on amine and/or carboxylate functional groups: (i) the deposition of polyethylimide (PEI), (ii) covalent binding with diethylenetriaminepentaacetic acid (DTPA), and (iii) conjugation with both PEI and DTPA, respectively. FT-IR, TGA, and DLS measurements confirmed that PEI or/and DTPA were successfully functionalized. The percentage of the free amine (−NH_2_) groups was also estimated. Increased magnetization values were found in case of PEI and DTPA-modified MNPs that stemmed from the adsorbed amine or oxygen ligands. Comparative UV–Vis studies for copper(II) ion removal from aqueous solutions were conducted, and the effect of time on the adsorption capacity was analyzed. The PEI-modified particles exhibited the highest adsorption capacity (164.2 mg/g) for copper(II) ions and followed the pseudo-second-order kinetics, while the polynuclear copper(II) complex Cu_x_(DTPA)_y_ was also able to be immobilized. The nanoadsorbents were quickly isolated from the solution by magnetic separation and regenerated easily by acidic treatment.

## 1. Introduction

The removal of heavy metals from wastewater has gained much attention because of their detrimental impact on ecological systems and human health [1,2]. In particular, copper, the third most abundant essential trace element in the human body, is frequently found in contaminated water [3,4]. However, high exposures of Cu^2+^ causes serious effects to humans, leading to neurodegenerative diseases [5], while enzymes, whose chemical behavior relay on sulfhydryl and amino groups, are strongly restrained by Cu^2+^ ions, which possess a high affinity for N and S comprising donor ligands [6,7]. Moreover, a considerable amount of heavy metals are able to coordinate various organic ligands in natural water or industrial effluents sourced from tanning or electroplating plants, hindering the self-purification capability of seas and rivers by chemical and biological degradation [8,9]. Hence, it is imperative to adopt efficient measures to handle the copper pollution in water effluents and biological systems, whether it is present as free ion or in the form of complexes. 

A variety of methods including electrolysis, catalytic degradation, ion exchange, membrane filtration, and adsorption have been proposed for the expelling of contaminants [10,11,12,13,14,15]. Most of them have been found to be limited due to cost, efficiency, and complexity. For instance, electrolysis processes often require higher operational costs, and the chemical precipitation may produce secondary wastes [16,17]. Of these techniques, adsorption is considered to be one of the most suitable choices because it uses cheap and non-pollutant materials that can be recycled and used easily on an industrial scale [18,19]. Nevertheless, conventional adsorbents show a poor renewal of the target metal ions from large volumes of solution due to a low binding capacity and a lack of active surface sites. In that way, the synthesis of a novel adsorbent with a large adsorptive surface area, a high adsorption capacity and a rapid separation for large volumes of solution is challenging [20].

Magnetic nanoparticles (MNPs) have been proposed as nanoadsorbents based on their combined magnetic and nanoscale properties. The synergistic effect between the core that can promote magnetic separation and the high specific surface area that can adsorb ions make them attractive for the removal of heavy metals [21,22,23,24,25]. Among magnetic nanoparticles, the use of Fe_3_O_4_ MNPs has been intensively examined due to their high magnetization. To further facilitate their adsorption capacity, surface modifications, including physical coating and covalent binding, have been explored to enable specific metal complexation [26,27,28]. However, the chemical stability for the oxidation of Fe_3_O_4_ is fairly poor, even if it is covered by an organic layer.

Recently, cobalt ferrite (CoFe_2_O_4_) nanoparticles have been explored as metal ion nanoadsorbents, owing to their high saturation magnetization, good chemical stability, and good corrosion resistance. Thus far, in all the reported cases, the same surface strategy has been adopted, as CoFe_2_O_4_ MNPs are covered by a silica shell that act as a platform to further graft amino or thiol functional groups. For example, thiol-functionalized silica-coated CoFe_2_O_4_ magnetic nanoparticles have been prepared to exclude Pb(II) or Hg(II) ions from water [29,30], while amino-functionalized CoFe_2_O_4_@SiO_2_ was designed by reverse co-precipitation for the removal of heavy metals [31]. However, the silica layer-mediated surface functionalization approach is complex, time consuming, and not favorable in large-scale applications. Importantly, the incorporation of silica into magnetic nanoparticles decreases the magnetic responsiveness of the nanocomposites due to the large total organic shell, which in some cases, has been found to reach a thickness of ~35 nm [31,32,33]. In addition, Si-O-Si bonds are prone to hydrolysis when exposed to harsh environments such as highly-saline conditions that are encountered in biological media [34,35,36].

In the present study, we report the formation of cobalt ferrite MNPs, and three different surface modifications were tested to detect their optimal performance as nanoadsorbents of copper(II) ions and copper complexes for aqueous solutions. Initially, fine cobalt ferrite MNPs coated with octadecylamine (ODA) and with a high saturation magnetization were solvothermally prepared and fully characterized. ODA, a linear aliphatic amine, was selected as a surfactant due to its ability, under specific synthetic conditions, to render free NH_2_ groups for further functionalization. Additionally, in order to provide a versatile interface the incorporation of chemical functional groups, poly(ethyleneimine) (PEI) and diethylenetriaminepentaacetic acid (DTPA) was tested. PEI is a relatively small polymer with amine donors, while DTPA, an octadentate aminopolycarboxylate agent that belongs to complexone molecules and is known for its high affinity complexation with metals. Adsorption capacity, the effect of reaction time on adsorption performance, and adsorption kinetics were investigated. Additionally, the metal stripping process by acid treatment was explored. Dealing with copper complexes, (Cu_x_(DTPA)_y_) was prepared and further used as a model complex to illustrate its adsorbance onto the MNPs’ surface. 

## 2. Materials and Methods

### 2.1. Chemical Reagents

All the reagents were of analytical grade and were used without any further purification. Iron(III) acetylacetonate (Fe(acac)_3_, ≥97.0%), octadecylamine (ODA, >90.0%), and N,N-Dimethyl formamide (DMF, >99.5%) were obtained from Fluka, (Bucharest, Romania); cobalt(II) acetylacetonate (Co(acac)_2_, ≥99.9%), chloroform (CHCl_3_, ≥99%), N-hydroxysuccinimide (NHS, ≥98.0%), poly(ethyleneimine) solution (PEI, ~25 kDa), diethylenetriaminepentaacetic acid (DTPA, ≥98.0%), and triethylamine (Et_3_N, ≥99.5%) were supplied by Aldrich, (Taufkirchen, Germany); ninhydrin was purchased from Merck, (Darmstadt, Germany); 1-(3-Dimethylaminopropyl)-3-ethylcarbodiimide hydrochloride, (EDC, >98.0%) was purchased from TCI, (Tokyo, Japan); copper(II) nitrate (Cu(NO)_2_, ≥99.9%) was obtained from Sinopharm Chemical Reagent Co., Ltd., (Shanghai, China); methanol (CH_3_OH, ≥99.9%) was supplied by Alfa Aesar, (Black freer, MA, USA); and ethanol (C_2_H_5_OH) was purchased from Bruggemann GmbH, (Heilbronn, Germany).

### 2.2. Preparation of Primary Cobalt Ferrite MNPs@ODA

The magnetic nanoparticles were prepared according to a solvothermal process that was previously reported by us [37]. Briefly, 1.8 mmol Fe(acac)_3_ and 0.9 mmol Co(acac)_3_ were mixed with 3.5 g of ODA, which acted in a triple role as surfactant, solvent, and reducing agent. The resulting solution was stirred thoroughly for 15 min, transferred into a 23 mL Teflon-lined stainless-steel autoclave, and then heated up with a stable rate of 4 °C/min until the temperature reached 200 °C, after which it remained stable for 24 h. After the 24 h reaction, the autoclave was left at room temperature, and CoFe_2_O_4_@ODA MNPs were isolated by centrifugation (5000 rpm/ethanol).

### 2.3. Surface Modifications of MNPs@ODA

#### 2.3.1. PEI Modification onto MNPs@ODA

Firstly, MNPs@ODA were dispersed in a CHCl_3_ (2 mg mL^−1^) forming solution A (5 mL), and PEI was dissolved in an H_2_O (20 mg mL^−1^) forming solution B (5 mL). Solutions A and B were mixed, and the resulting mixture was shaken for 3 h. At the end, the MNPs were moved into the aqueous phase, and the organic solvent was slowly evaporated. MNPs@ODA covered with PEI (~25 kDa), namely MNPs@ODA@PEI, were isolated by centrifugation (5000 rpm/ethanol).

#### 2.3.2. Direct Coupling of DTPA onto MNPs@ODA

The direct coupling of DTPA onto the surface of MNPs@ODA was achieved with a modified method [38], where 0.3 mmoL of DTPA was dissolved in 5 mL of H_2_O and EDC (0.3 mmoL), NHS (0.3 mmoL), and Et_3_N (50 μL) were added. The resulting solution was mixed with 20 mg of MNPs@ODA dispersed in 5 mL of CHCl_3_. The mixture was shaken vigorously for 24 h, and MNPs@ODA@DTPA were isolated by centrifugation (5000 rpm/ethanol).

#### 2.3.3. Direct Coupling of DTPA onto MNPs@ODA@PEI

For the deposition of DTPA onto MNPs@ODA@PEI, 0.4 mmoL of DTPA were dissolved in 10 mL of H_2_O, and EDC (0.4 mmoL) with NHS (0.4 mmoL) were added. In the resulting solution, 20 mg of MNPs@ODA@PEI were inserted. After vigorously shaking for 24 h, MNPs@ODA@PEI@DTPA were isolated by centrifugation (5000 rpm/ethanol).

### 2.4. Ninhydrin Colorimetric Assay

The concentration of the free amine groups on the MNPs’ surface was determined by a ninhydrin colorimetric assay [39]. The method is based on the formation of Ruhemann’s purple complex, which is indicative of the reaction between amino groups and ninhydrin. The presence of the complex can be detected by UV–Vis at 600 nm. For the calibration curve, aliquots of ODA (0.1–0.6 mL, 0.25 mg mL^−1^) in dimethylformamide (DMF) were inserted into a series of tubes. To each tube, ninhydrin solution in MeOH (0.06 m, 0.7 mL, 10.7 mg mL^−1^) was added, and the mixture was mixed well and heated at 100 °C for 5 min. After heating, the tubes were left to cool, and the contents were transferred to a 5 mL volumetric flask and diluted with DMF (2 mL), and then the UV–Vis absorbance was measured. A solution of particles in DMF (0.25 mg mL^−1^) was prepared accordingly, and 0.4 mL of the sample stock solution was transferred into boiling tubes with a ninhydrin solution (0.7 mL) by following the same procedure described above, and UV–Vis measurements of the formed complex were performed. DMF was chosen because it leads to good dispersions of particles.

### 2.5. Batch Adsorption Experiments and Copper(II) Detection

Adsorption experiments were performed at pH 7 and at room temperature. Each batch experiment was conducted three times, and the data shown are the average values. Adsorption experiments were performed by mixing aqueous solutions (1 mL) of the samples (1 mg mL^−1^) with a copper nitrate solution (5 mM, 1 mL) for different reaction times (15 and 30 min; 1, 2, 4, 8, and 24 h). The detection of copper was based on the method of Wen et al. [40] and conducted as follows: 1 mL of the resulting solution was centrifuged (7500 rpm, 12 min), and the particles were magnetically separated. The supernatant was analyzed by UV–Vis (200–400 nm). In particular, 250 μL of the supernatant were diluted with 200 μL of water, and 500 μL of an aqueous PEI solution (0.94 mg mL^−1^) were added. From the resulting solution, 400 μL were diluted in water in order to obtain a volume of 2 mL for each measurement.

### 2.6. Copper Complex (Cu_x_(DTPA)_y_)

#### 2.6.1. Preparation of the Complex *(Cux(DTPA)y)*

In 20 mL of MeOH, 0.6 mmol of DTPA were dissolved, and 3 mmol of NaOH were added. After 30 min of stirring, the resulting solution was added dropwise to 0.6 mmol (200 mg) of CuCl_2_·2H_2_O dissolved in CH_3_CN (10 mL). The reaction mixture was stirred and heated (60 °C) for an additional 1 h, then was filtered and left for slow evaporation. The blue solid was precipitated after two days and filtered. 

#### 2.6.2. Immobilization of *(Cu_x_(DTPA)_y_)* onto MNPs@ODA@PEI

In 5 mL of water (pH 7), 100 mg of (Cu_x_(DTPA)_y_) were dissolved, forming solution A. Then, 10 mg of MNPs@ODA@PEI were dispersed in 5 mL of CHCl_3_, forming solution B. Solutions A and B were mixed and stirred for 3 h at 37 °C. The final product was isolated by centrifugation (5000 rpm/ethanol).

#### 2.6.3. UV–Vis Studies of MNPs@ODA@PEI@*(Cu_x_(DTPA)_y_)*

An aqueous solution of (Cu_x_(DTPA)_y_) (0.5 mg mL^−1^) was prepared and mixed with 10 mg of MNPs@ODA@PEI for different reaction times (30, 60, and 120 min). After the immobilization, MNPs@ODA@PEI@(Cu_x_(DTPA)_y_) were magnetically separated from the aqueous solutions, and the residual concentrations of metal ions in the aliquots were determined by UV–Vis analysis (400–900 nm).

### 2.7. Characterization Techniques

XRD measurements were performed with a two-cycle diffractometer (40 kV, 30 mA, Cu-K_a_ radiation), Rigaku Ultima, Austin, TX, USA. TEM images were obtained with a transmission electron microscope (JEOL 100 CX, Tokyo, Japan). The elemental composition of the samples was estimated with a JEOL 840A (Tokyo, Japan) scanning electron microscope (SEM). Hydrodynamic diameters and ζ-potentials were determined via electrophoretic measurements that were carried out at 25 °C by means of a NanoZetasizer DLS, (Nano ZS Malvern apparatus, Worcestershire, England, UK). Fourier transform infrared spectroscopy (400–4000 cm^−1^) was recorded with a Nicolet FT-IR 6700 spectrometer, (Thermo Electron, Waltham, MA, USA), with samples prepared as KBr pellets. Thermogravimetric analysis (TGA) was performed with a SETA-RAM SetSys-1200 and carried out in the range from room temperature to 900 °C at a heating rate of 10 °C min^−1^ under an N_2_ atmosphere. UV–Visible measurements were carried out with a spectrophotometer, (2001 Hitachi, Kagoshima, Japan). Magnetic measurements were performed with vibrating sample magnetometer (VSM) (P.A.R. 155).

## 3. Results and Discussion

Initially, uniform cobalt ferrite MNPs coated with octadecylamine (ODA) were solvothermally prepared according to our previous work [37]. For the synthesis of the magnetic nanoadsorbents, three different surface modifications were conducted and compared regarding their adsorption capacity: (i) PEI has attracted great attention due to its particular characteristics of structure, branched internal cavity, and abundant terminal amines [41,42]. In general, an amino-group is commonly used to functionalize nanomaterials and has demonstrated an outstanding ability to remove a wide variety of heavy metal ions and biomolecules due to the strong complex capability of the lone pair electron of nitrogen atoms [43,44,45,46]; (ii) DTPA is a labile chelating agent that contains three nitrogen atoms bound to five carboxylate groups [47,48]. As an artificial amino acid, it is known as complexone and is widely studied and used for the mobilization of heavy metals and radionuclides from contaminated soils [49,50,51]; (iii) the employment of both PEI and DTPA was expected to provide mixed functional groups (amines and carboxylates) and to increase the adsorption of metal ions. Compared to other studies on CoFe_2_O_4_ MNPs that have been based on silica functionalization, the direct modification of these agents ensures a prompt response to metal ion removal [29,30,31,32].

### 3.1. Structural and Surface Characterization

**Figure 1:**
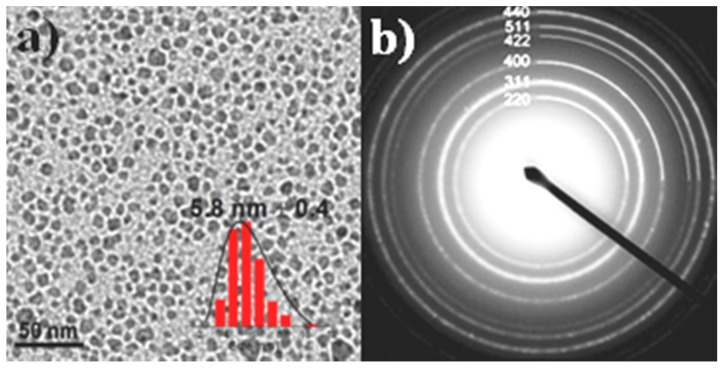
(**a**) TEM image and particle size distribution histogram (inset) of the primary magnetic nanoparticles coated with octadecylamine (MNPs@ODA) and (**b**) the electron diffraction pattern of the cobalt ferrite particles.

The XRD pattern of the primary MNPs disclosed the diffraction peaks of the spinel structure of the cobalt ferrite (JCPDS/file No. 22-1086), with crystalline size, calculated from Scherrer equation, equal to 6.5 nm (Figure S1). From TEM imaging (Figure 1a), the size was found to be 5.8 nm ± 0.4 nm, which was in good agreement with XRD results, while the EDS measurement revealed an iron-rich cobalt ferrite Co_0.76_Fe_2.24_O_4_.

**Figure 2:**
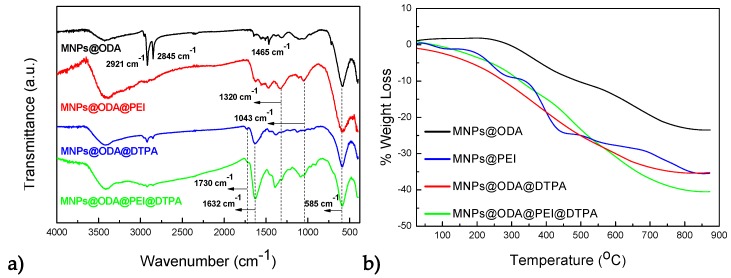
(**a**) FT-IR spectra of the primary MNPs@ODA together with the three surface-modified samples and (**b**) the weight loss of the organic amount under argon atmosphere of the four samples.

The successful modifications with PEI and/or DTPA on the surface of the primary particles (MNPs@ODA) were observed by FT-IR spectra and are shown in Figure 2a. All samples revealed absorption bands in a good agreement with the pure organic molecules, which are presented as reference in Figure S2. In the initial MNPs@ODA, the asymmetric (2921 cm^−1^) and symmetric (2845 cm^−1^) stretching vibrations of the methylene groups and the characteristic peak of ODA at 1465 cm^−1^ were present. For MNPs@ODA@PEI, all the characteristics peaks of PEI were found, while the C-N stretch vibration appeared shifted at 1320 instead of 1306 cm^−1^ of the pure PEI (Figure S2) due to the interaction with the particles’ surface. In the case of MNPs@ODA@DTPA and MNPs@ODA@PEI@DTPA, the weak peaks at 1730 cm^−1^ were ascribed to the protonated carboxylic groups, while the strong peaks at 1632 cm^−1^ were attributed to the deprotonated asymmetric stretching vibration ν_as_(CO_2_), indicating that DTPA was partially deprotonated. Finally, in all samples, the typical low frequency band at around 585 cm^−1^ was caused by the motion of oxygen with respect to the cations in the octahedral (O_h_) sites, characteristic of the spinel structure [52].

The presence of the amine groups on the MNPs’ surface was further detected with the ninhydrin colorimetric assay. At *λ_max_* ~600 nm, the appearance of Ruhemann’s purple complex verified the synthesis of particles with aminated surfaces (Figure S3). The free –NH_2_ for the initial MNPs@ODA was found equal to 0.24 mg/mg_MNPs_, while in the case of MNPs@ODA@PEI, the free –NH_2_ was drastically increased up to 0.42 mg/mg_MNPs_. For MNPs@ODA@DTPA and MNPs@ODA@PEI@DTPA, the amine groups were 0.18 and 0.28 mg/mg_MNPs_, respectively, indicating that a number of the primary amine groups were still present on the surface of the MNPs. 

The amount of the organic coating was quantitatively recorded by thermogravimetric analysis (TGA); see Figure 2b. For all samples, the weight loss at temperatures below 200 °C was attributed to the release of moisture on the surface of samples. At higher temperatures (>220 °C), the decomposition of the organic coating occurred at multiple steps. The total organic amount for each sample was estimated to be 23% (MNPs@ODA), 35% (MNPs@ODA@PEI), 35% (MNPs@ODA@DTPA), and 41% (MNPs@ODA@PEI@DTPA). 

**Figure 3:**
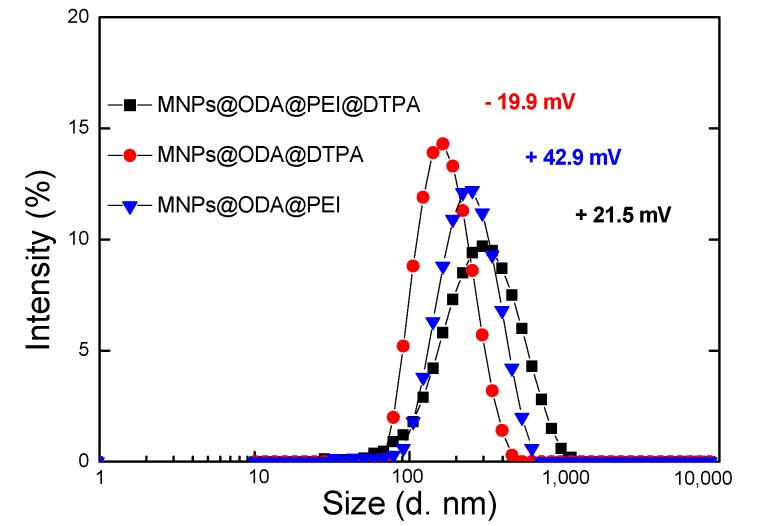
Hydrodynamics diameters and ζ-potentials of the three surface-modified samples.

The modified MNPs were homogeneously dispersed in water. The hydrodynamics diameters were found to be equal to 177, 159, and 215 nm for MNPs@ODA@PEI, MNPs@ODA@DTPA, and MNPs@ODA@PEI@DTPA, respectively (Figure 3). In addition, the ζ-potential of their aqueous suspensions at neutral pH were positive for MNPs@ODA@PEI (+42.9 mV) and MNPs@ODA@PEI@DTPA (+21.5 mV) due to the amine groups, while the same for MNPs@ODA@DTPA was found to be negative (−19.9 mV), indicating the presence of deprotonated carboxyl groups (Figure 3).

### 3.2. Magnetic Studies

**Figure 4:**
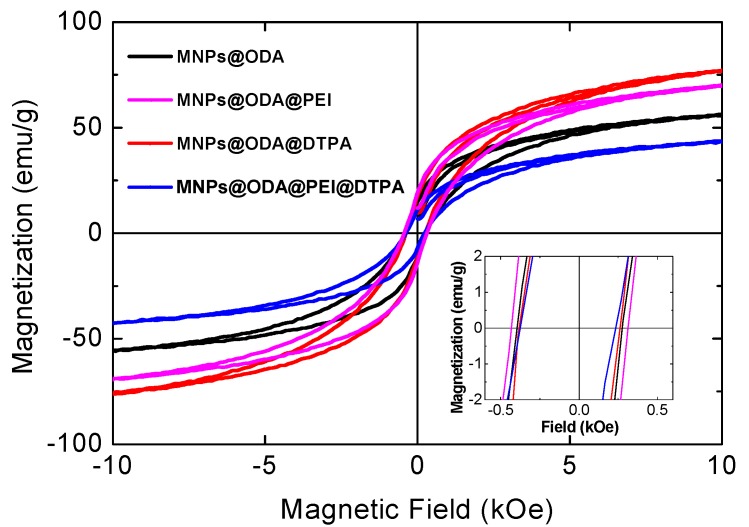
Room-temperature hysteresis loops recorded by vibrating sample magnetometry (VSM).

Magnetic properties were examined at room temperature by measuring the magnetization as a function of an external field up to 10 kOe (Figure 4). Remarkably, even though the magnetization of the primary cobalt ferrite MNPs@ODA was found moderate at 45 emu/g, after the surface modifications, it was increased up to 70 emu/g for MNPs@ODA@PEI and up to 77 emu/g for MNPs@ODA@DTPA. For both samples, the increased magnetization stemmed from the adsorbed amine or oxygen ligands (σ-donor), which took the position of the oxygen vacancies and controlled the symmetry and spin–orbit coupling of the surface metal ions more closely to that of the core [53]. This phenomenon assisted in the compression of the surface spin disorder layer (also called the ‘dead layer’) on the nanoparticles’ surface by repairing the broken and dangling bonds of Fe–O/Co-O, consequently increasing magnetization. In so, these values were relatively higher than those that have been reported for silica-modified CoFe_2_O_4_ nanoparticles with similar sizes [29,30,31,32]. However, the third modification route (MNPs@ODA@PEI@DTPA) led to reduced magnetization (44 emu/g) in accordance to the larger organic layer detected from the TGA results. For that sample, we speculate that DTPA did not coordinate with the metal cores due to the distance from the surface. Regarding coercivity, the values were found without significant differences ranging from 300 to 370 Oe, indicating the soft-magnetic behavior of the systems before and after the surface reconstructions. The absolute values of magnetization (M_S_) were estimated from the effective mass of the MNPs in connection with the TGA results (Table S1).

### 3.3. Absorption Spectroscopy Studies

**Figure 5:**
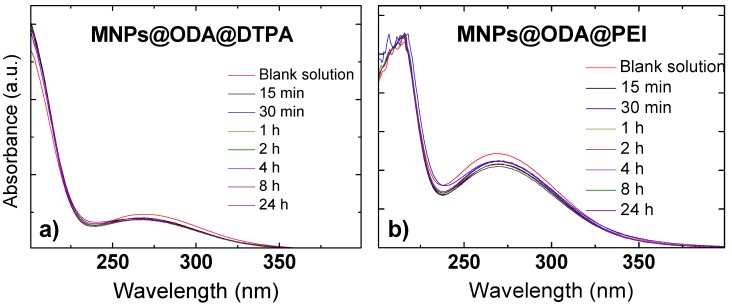
UV–Vis titration of the blank solution (Cu(NO_3_)_2_) with (**a**) (MNPs@ODA coupled with diethylenetriaminepentaacetic acid) MNPs@ODA@DTPA and (**b**) (MNPs@ODA coupled with polyethylimide) MNPs@ODA@PEI.

The functionalized samples, MNPs@ODA@PEI, MNPs@ODA@DTPA, and MNPs@ODA@PEI@DTPA, were applied as adsorbents to remove copper ions from water. The UV–Vis titration absorption spectra of the samples in an aqueous solution are provided in Figure 5 and Figure S4, while the detection of Cu^2+^ was based on the method of Wen et al. [40]. Firstly, the absorbance of an aqueous solution of copper(II) nitrate (5 mM) was measured in the absence of MNPs (blank solution). Then, aqueous solutions of MNPs (1 mg/mL) were added and left for different time intervals. At the end, the MNPs were magnetically separated, and the absorbance of the supernatants was measured again. From the absorbance, the concentration of the remaining copper(II) nitrate was detected to enable the calculation of adsorption capacities (mg/g) of the samples with the following equation:

(1) $$q_{t}=\frac{\left(C_{0}-C_{t}\right)V}{m}$$

where *C*_0_ and *C_t_* (mg/L) are the initial concentrations of the ions (5 mM) and at time *t*, respectively; *V* (mL) is the volume of the adsorbate (1 mL); and *m* (mg) is the mass of the adsorbent (1 mg).

The metal uptake capacities of the samples revealed that MNPs@ODA@PEI (164.2 mg/g) exhibited a superior sorption efficiency compared to MNPs@ODA@DTPA (29.4 mg/g) and MNPs@ODA@PEI@DTPA (40.4 mg/g). This value can be considered among the best of those previously reported for nanoabsorbents [25,31,54], while can be attributed to the polymeric branched nature of PEI and the greater amount of amino groups that were present. Even though the surface of the PEI-modified particles was strongly positive charged (+42.9 mV), suggesting that amino groups were protonated to -${\mathrm{NH}}_{3}^{+}$, a sufficient percentage of -NH_2_ was available. Therefore, the high adsorption of MNPs@ODA@PEI was derived from the complexation of amine groups with Cu^2+^ and from the branched structure of PEI that permitted the intercalation of the metals.

**Figure 6:**
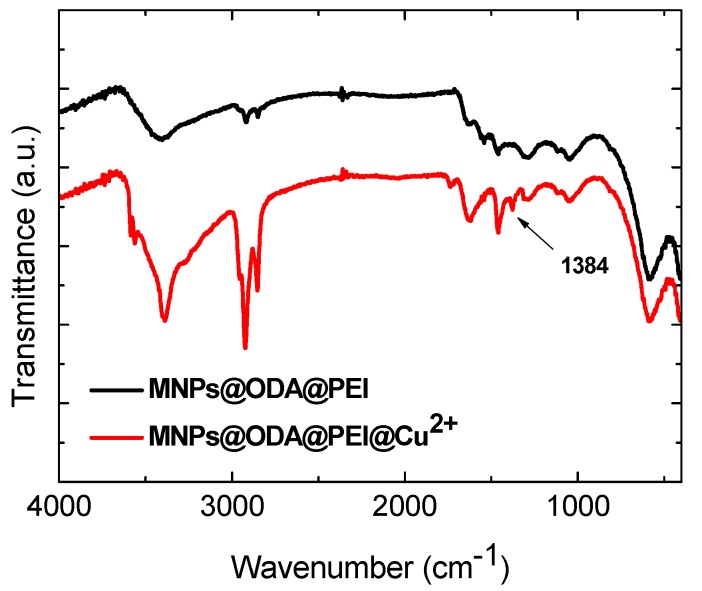
Indicative FT-IR spectra of MNPs@ODA@PEI before and after the adsorption of copper ions.

The FT-IR spectra of the samples before and after adsorption are presented in Figure S5. In all cases, the spectra showed that the peaks of the primary particles were slightly shifted, suggesting that the functional groups chemically reacted with copper. Additionally, in all samples, the peak at 1384 cm^−1^ stemmed from residual ${\mathrm{NO}}_{3}^{-}$ ions, which were also adsorbed on the surface in order to counterbalance the positive surface of the particles and complete the coordination sphere of the copper ions (Figure 6) [55]. The nitrate ions derived from the initial precursor, copper(II) nitrate, which was used in the adsorption experiments.

### 3.4. Adsorption Kinetics

**Figure 7:**
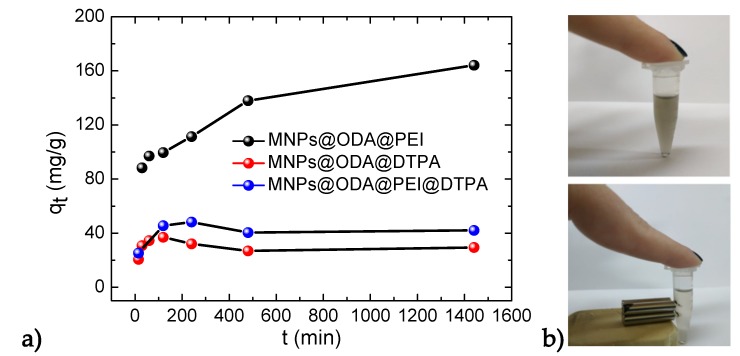
(**a**) Effect of time on the adsorption process and (**b**) magnetic separation by external magnet.

The adsorption tendencies of the two samples, MNPs@ODA@DTPA and MNPs@ODA@PEI@DTPA, were found similar, as they rose sharply within the initial 60 min, and then they remained almost steady even when the contact time was extended to 1400 min. In contrast, the adsorption capacity of the PEI-modified particles (MNPs@ODA@PEI) constantly rose as time increased. Moreover, by using a commercial magnet, the MNPs were rapidly separated from the solution within a few seconds (Figure 7b). 

To evaluate the kinetic process, the pseudo-first-order and the pseudo-second-order models were tested. The first order model can be generally expressed as [56]:

(2) $$\frac{dq_{t}}{dt}=K\left(q_{e}-q_{t}\right)$$

The integral form of this equation is given as follows:

(3) $$\log(q_{e}-q_{t})=\log q_{e}-k_{1}t/2.303$$

where *q_e_* is the equilibrium adsorption capacity of the adsorbent (mg g^−1^), *q_t_* is the adsorption capacity (mg g^−1^) when the contact time is *t*, and *K* and *k*_1_ are the rate constants of the pseudo first-order model. On the other hand, the pseudo-second-order model can be expressed as [57]:

(4) $$\frac{dq_{t}}{dt}=k_{2}{\left(q_{e}-q_{t}\right)}^{2}$$

**Figure 8:**
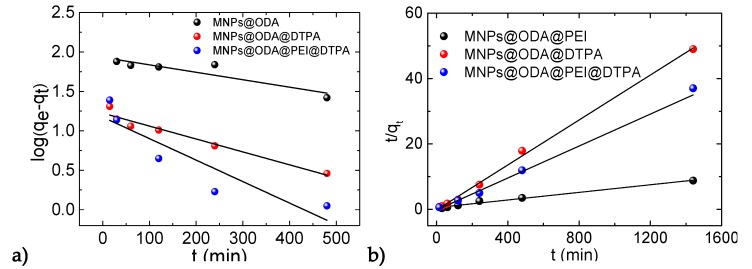
Pseudo-first-order (**a**) and pseudo-second-order models (**b**) of all samples.

The integral form of this equation is given as follows:

(5) $$\frac{t}{q_{t}}=\frac{1}{k_{2}q_{e}^{2}}+\frac{t}{q_{e}}$$

where *q_e_* is the equilibrium adsorption capacity of the adsorbent (mg g^−1^), *q_t_* is the adsorption capacity (mg g^−1^) when the contact time is *t*, and *k*_2_ is the rate constant of the pseudo second-order adsorption model. The linear fitting degrees of the two models are given in Figure 8. 

In Table 1, the correlation coefficients estimated from the two models are presented. The q_e_(cal) values based on the second model were 171.8, 29.1, and 40.9 mg/g, which were close to the experimental values q_e_(exp). On the other hand, the calculated qe values obtained by the first model were not in agreement with the experimental q_e_ values, denoting that the adsorption of Cu(II) ions did not follow pseudo-first-order kinetics.

The values of the correlation coefficients of the pseudo-second-order model for Cu(II) ions were 0.984, 0.997, and 0.984. All of them were higher than the values obtained by the pseudo-first-order model. This clearly indicated that the applicable adsorption kinetics followed the pseudo-second-order model. Therefore, the overall rate of the adsorption process was governed by chemisorption rather than mass transport [58].

The regeneration of the adsorbent, i.e., the restoration of adsorption capability, is a crucial factor in practical applications. Through acid treatment (15 min, 0.1 M HCl solution), the particles were “cleaned” and isolated through aqua washes and centrifugation. From FT-IR spectroscopy (Figure S6), the effective regeneration of the particles was evidenced, as the peak at 1384 cm^−1^ vanished and the spectrum of the regenerated MNPs@ODA@PEI resembled the initial spectrum.

### 3.5. Immobilization of the (Cu_x_(DTPA)_y_) onto MNPs’ Surface 

The complexone DTPA was selected as ligand to form the (Cu_x_(DTPA)_y_) complex. Details about the chemical structure of the synthesized (Cu_x_(DTPA)_y_) were gathered by FT-IR spectroscopy (Figure S7) and magnetic susceptibility (χ_g_) measurements. In Figure S8, the proposed chemical structure of the complex is given in agreement with the literature data [59,60]. 

**Figure 9:**
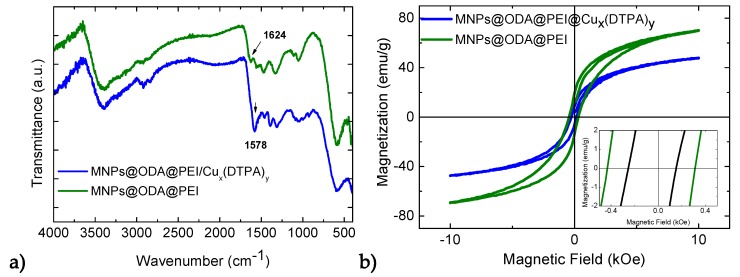
(**a**) FT-IR spectra of the nanoparticles with (blue line) and without (green line) the presence of the complex and (**b**) the hysteresis loops of the MNPs before and after the functionalization with the complex.

Based on the fact that the modified MNPs@ODA@PEI demonstrated the higher adsorption capacity, we further used these MNPs as nanoabsorbents for the model complex (Cu_x_(DTPA)_y_). In this experiment, FT-IR, SEM, and VSM analyses were performed in the solid state to probe the successful entrapment of the complex. SEM analysis confirmed the presence of copper (~4%) (Figure S9). The IR spectra before and after the absorption of the complex are given at Figure 9a. The strong peak at 1578 cm^−^^1^ certified the presence of the asymmetric stretching vibration ν_as_(CO_2_) of the fully deprotonated form of DTPA and was in accordance with the IR spectrum of (Cu_x_(DTPA)_y_) (Figure S7). In addition, the reduced magnetization of MNPs@ODA@PEI from 70 to 48 emu/g also evidenced of the copper complex deposition onto the MNPs’ surface (Figure 9b). Moreover, the observed reduced coercivity, at around 200 Oe, arose from the reduced surface anisotropy as a result of the presence of copper ions close to the surface of the MNPs. The paramagnetic ions could interact via exchange interactions with the metal centers of the surface, leading to a better alignment of the spins. Such change certainly affected the surface anisotropy and, consequently, the coercivity of the particles [61]. 

**Figure 10:**
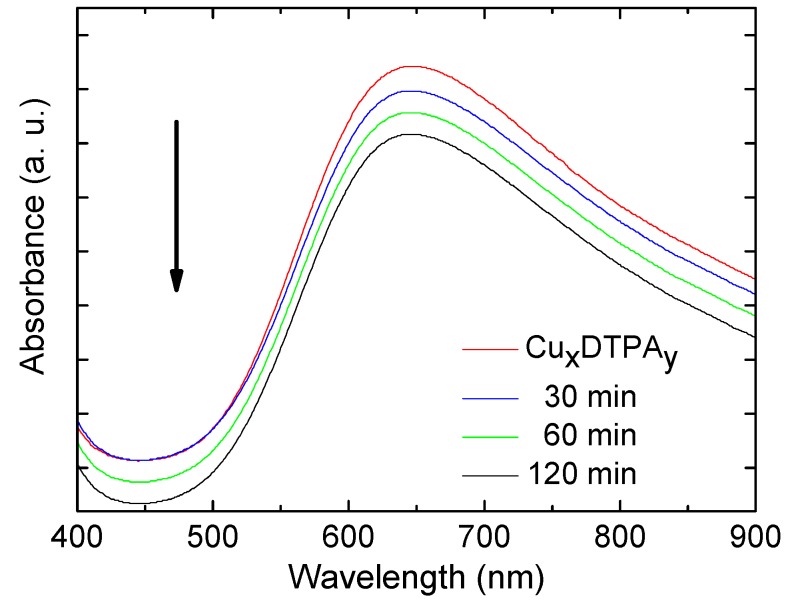
UV–Vis spectra of the copper complex in an aqueous solution with the absorbance decay curves of the supernatants.

The successful immobilization of the (Cu_x_(DTPA)_y_) onto the MNPs’ surface was followed by UV–Vis studies (Figure 10). The absorbance spectrum of the complex dissolved in water was located at 645 nm. After the addition of MNPs@ODA@PEI into the solution and their magnetic extraction at different time intervals, the measurement was repeated for the supernatants. The results revealed time-dependent behavior, as the absorbance of the supernatants gradually decreased and confirmed the removal of different portions of the complex. An accurate estimation of adsorption capacity (q_t_) was inadequate, as the initial concentration (C_0_) (Equation (1) could not be calculated due to the lack of the molecular weight of the copper complex, as the precise chemical formula was unknown. However, by a rough estimation based on the complex’s absorbance, the magnitude q_t_ values were found to 1.4 mg/g (30 min), 3.0 mg/g (60 min), and 4.2 mg/g (120 min).

## 4. Conclusions

Seeking to find new nanoadsorbents, fine cobalt ferrite nanoparticles, known for their chemical stability in harsh conditions, were prepared with an amine coating that could be further utilized for functionalization. The modification of the primary MNPs@ODA was achieved by three surface-chemistry routes with PEI or/and DTPA. The alteration of the chemical composition of the surface of the MNPs resulted in different adsorption capabilities for copper(II) ion and copper complex removal from aquatic solutions. Neither the covalent binding of DTPA nor the combination of DTPA with PEI on the surface of the MNPs were as efficient as the PEI-modified particles, which presented the highest adsorption capacity (164.2 mg/g) within 60 min. Using these magnetic nanoprobes, copper ions could be collected and separated with a commercial magnet, while the nanoparticles could be well-regenerated for reuse. In addition to copper(II) removal, the PEI-modified particles were capable of adsorbing the (Cu_x_(DTPA)_y_) complex onto their surface. Overall, compared to other studies, PEI-modification can be considered as a simple chemical approach that is economic and effective in imparting diverse surface properties to magnetic nanoadsorbents without the loss of their magnetic performance.

## Figures and Tables

**Table 1:** Parameters for the pseudo-first-order and pseudo-second-order models.

Pseudo-First-Order			Pseudo-Second-Order				
Sample	q_e_(cal) (mg/g)	k_1_ (1/min)	R^2^	q_e_(cal) (mg/g)	k_2_ (mg/gmin)	R^2^	q_e_(exp) (mg/g)
MNPs@ODA@PEI	85.1	0.245	0.774	171.8	0.011	0.984	164.2
MNPs@ODA@DTPA	16.6	0.144	0.948	29.1	0.007	0.997	29.4
MNPs@ODA@PEI@DTPA	14.9	0.115	0.772	40.9	0.003	0.984	40.4

## Supplementary Materials

The following are available online at https://www.mdpi.com/1996-1944/13/7/1537/s1, Figure S1. XRD pattern of the cobalt ferrite MNPs@ODA. Figure S2. FT-IR spectra of the pure surfactants. Figure S3. Ninhydrin colorimetric analysis of the primary MNPs@ODA with the three surface modified samples. Figure S4. UV–Vis titration of blank solution (Cu(NO_3_)_2_) with MNPs@ODA@PEI@DTPA. Figure S5. FT-IR spectra of the MNPs@ODA@PEI@DTPA and MNPs@ODA@DTPA before and after the adsorption of copper ions. Figure S6. FT-IR spectra of the regenerated MNPs@ODA@PEI. Figure S7. FT-IR spectra of the ligand DTPA and the copper complex. Figure S8. Proposed structure of the copper complex. Figure S9. SEM imaging and elemental analysis of the MNPs@ODA@PEI@(Cu_x_(DTPA)_y_). Table S1. Corrected magnetization values according to the percentage of the organic coatings.
