# Supplementary material for: Diverse Surface Chemistry of Cobalt Ferrite Nanoparticles to Optimize Copper(II) Removal from Aqueous Media
Source: Materials (Basel). 2020 Mar 27;13(7):1537. doi: 10.3390/ma13071537 (PMC7177944; doi:10.3390/ma13071537)
Supplement: Supplementary file 1 [file materials-13-01537-s001.pdf]

materials

MDPI

Supplementary Materials: Diverse Surface Chemistry of Cobalt Ferrite Nanoparticles to Optimize Copper(II) Removal from Aqueous Media

Kosmas Vamvakidis , Theodora-Marianna Kostitsi, Antonis Makridis and Catherine Dendrinou-Samara *

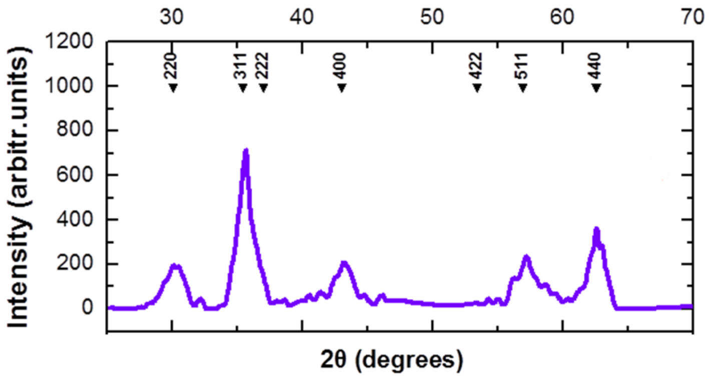

Figure S1. XRD pattern of the cobalt ferrite MNPs@ODA. All the peaks are well matched with the standard JCPDS data for $CoFe_2O_4$ (file No. 22-1086).

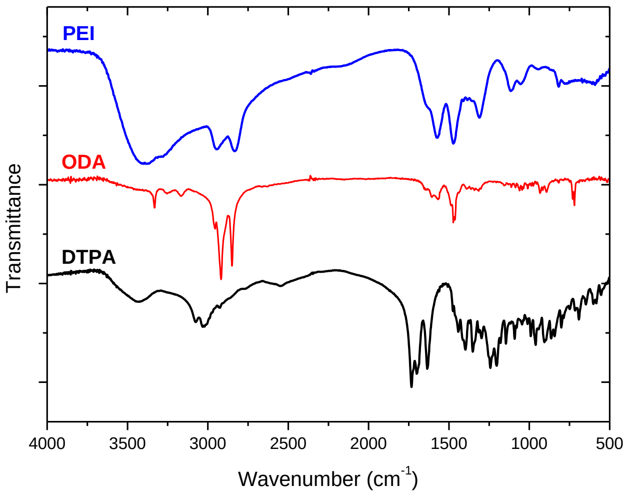

Figure S2. FT-IR spectra of the pure surfactants.

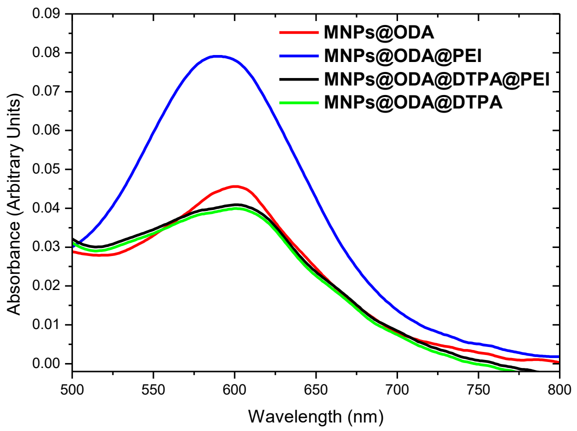

Figure S3. Ninhydrin colorimetric analysis of the primary MNPs@ODA with the three surface modified samples.

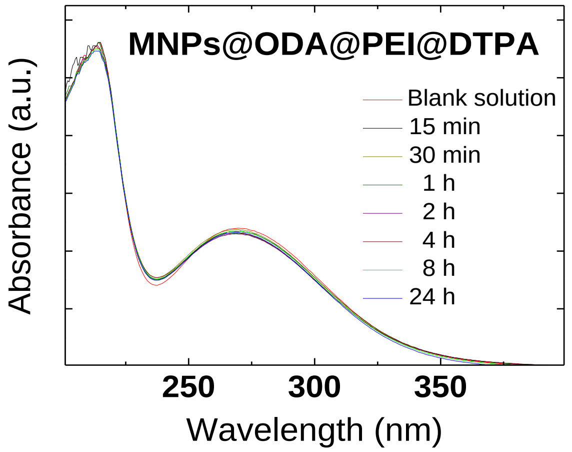

Figure S4. UV-Vis titration of blank solution [Cu(NO3)2] with MNPs@ODA@PEI@DTPA.

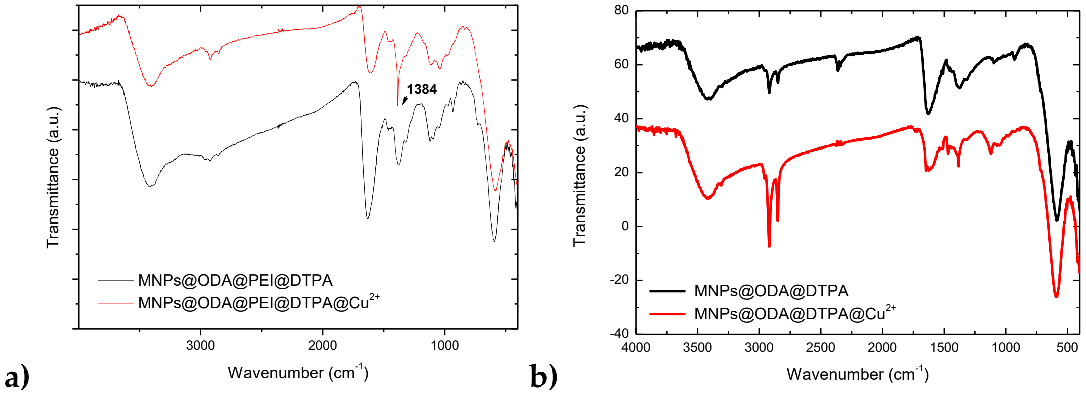

Figure S5. FT-IR spectra of the MNPs@ODA@PEI@DTPA and MNPs@ODA@DTPA before and after the adsorption of copper ions.

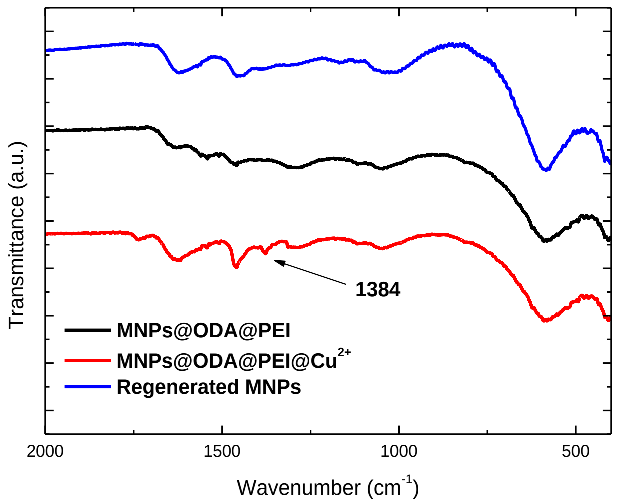

Figure S6. FT-IR spectra of the regenerated MNPs@ODA@PEI after three washes.

Some of the characteristics IR bands of DTPA (Figure 6S) are the double peak at 1733 and 1701 cm^{-1} stemming from the asymmetric stretching of carboxyl groups, as well as the symmetric band appearing at 1635 cm^{-1}. In addition, the peaks at 1240 and 1397 cm^{-1} arise from the stretching of C-O and C-N, respectively. In the spectrum of the complex the stretching peaks of the protonated carboxylic groups are not appeared and only the symmetric (1400 cm^{-1}) and the antisymetric (1610 cm^{-1}) peaks of the deprotonated –COO^- are present (Figure 6S). These findings designate bidentate coordination of the DTPA towards copper ions. Additionally, the magnetic moment of the complex was found equal to 0.77 μ_B, suggesting the existence of exchange interactions among closed metal ions and in so the formation of a polynuclear copper complex.

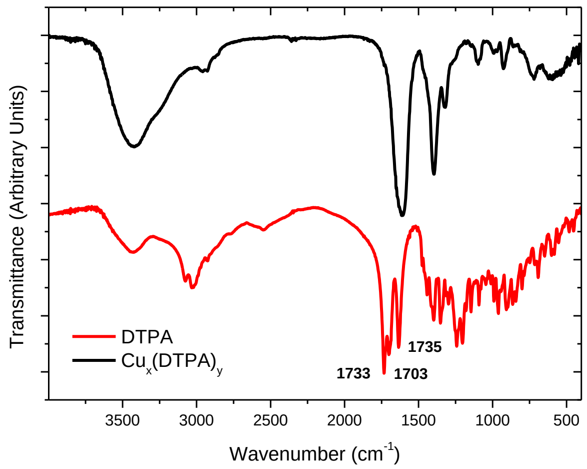

Figure S7. FT-IR spectra of the ligand DTPA and the copper complex.

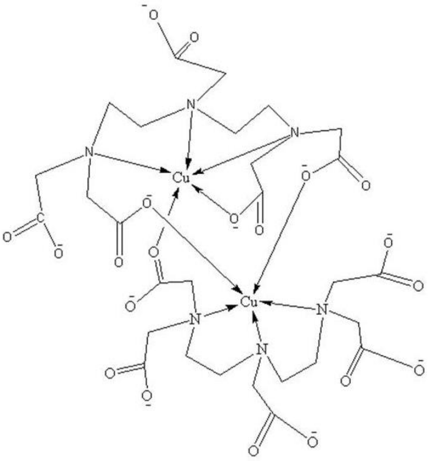

Figure S8. Proposed structure of the copper complex.

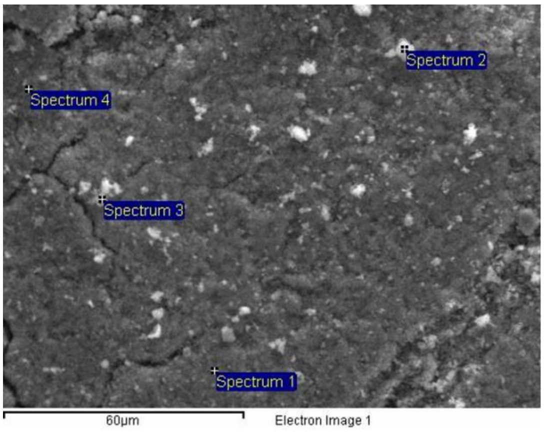

Processing option : All elements analysed (Normalised)

Spectrum	In stats.	Fe	Co	Cu	Total
Spectrum 1	Yes	73.88	24.05	2.07	100.00
Spectrum 2	No	82.73	19.60	-2.32	100.00
Spectrum 3	Yes	72.14	21.45	6.41	100.00
Spectrum 4	Yes	73.69	22.99	3.32	100.00
Mean		73.24	22.83	3.93	100.00
Std. deviation		0.96	1.30	2.24	
Max.		73.88	24.05	6.41	
Min.		72.14	21.45	2.07	

All results in weight%

Figure S9. SEM imaging and elemental analysis of the MNPs@ODA@PEI@($Cu_x(DTPA)_y$).

Table S1. Corrected magnetization values according to the percentage of the organic coatings.

Sample	Ms (emu/g)	% TGA
MNPs@ODA	71	23
MNPs@ODA@PEI	88	35
MNPs@ODA@DTPA	98	35
MNPs@ODA@PEI@DTPA	75	41
MNPs@ODA@PEI@$Cu_x(DTPA)_y$	77	38

© 2020 by the authors. Licensee MDPI, Basel, Switzerland. This article is an open access article distributed under the terms and conditions of the Creative Commons Attribution (CC BY) license (http://creativecommons.org/licenses/by/4.0/).
